# Long-term outcomes of refractory central venous occlusive disease treated by stent deployment in patients undergoing maintenance hemodialysis

**DOI:** 10.1080/0886022X.2025.2463579

**Published:** 2025-02-16

**Authors:** Yuqin Xiong, Yan Wang, Xiaoqin He, Yi Ruan, Yue Wen, Yang Yu, Ping Fu

**Affiliations:** ^a^ Department of Nephrology, Institute of Kidney Diseases, West China Hospital of Sichuan University, Chengdu, PR China; ^b^ Innovation Institute for Integration of Medicine and Engineering, West China Hospital, Sichuan University, Chengdu, PR China; ^c^ Hemodialysis Center, the People’s Hospital of Leshan Central District, Leshan, PR China; ^d^ Division of Radiology, West China Hospital of Sichuan University, Chengdu, China

**Keywords:** Hemodialysis, central venous occlusive disease, stent deployment, vascular access, mortality

## Abstract

**Objectives:**

To investigate the long-term outcomes of stent deployment in the treatment of refractory central venous occlusive disease (CVOD) in patients undergoing maintenance hemodialysis (MHD).

**Methods:**

MHD patients who were successfully treated with stenting for symptomatic CVOD that was resistant to balloon angioplasty alone were consecutively included in this retrospective study. The primary (PPR) and assisted (APR) patency rates of the central vein and hemodialysis vascular access (VA), reintervention, and survival rates after stenting were followed. Multivariate logistic regression analyses were conducted to determine the factors influencing VA abandonment and mortality.

**Results:**

The cohort comprised 65 patients (52.3% male) aged 61.5 ± 13.5 years, with a mean dialysis vintage of 54.7 ± 39.1 months. During 40 (20–54) months of follow-up, symptomatic CVOD recurred in 32 (49.2%) patients, accounting for 51 secondary angioplasties, including 34 stenting procedures. The PPR and APR at 12, 24, 36, 48, and 60 months were 81%, 52%, 47%, 41%, and 41% and 98%, 98%, 82%, 82%, and 82%, respectively. VA abandonment was noted in 10 (15.4%) patients. Six (9.2%) and 17 (26.2%) patients died due to cardiovascular conditions and all causes, respectively. The number of secondary stenting procedures was significantly associated with decreased VA abandonment [odds ratio (OR) = 0.089, 95% confidence interval (CI): 0.008–0.992, *p* = 0.049] and all-cause mortality (OR = 0.104, 95% CI: 0.011–0.947, *p* = 0.045).

**Conclusions:**

Angioplasty with stenting is an effective and promising strategy for MHD patients with refractory CVOD.

## Introduction

Central venous stenosis or occlusion, which is referred to as central venous occlusive disease (CVOD), is a frequent (25%–40%) and intractable problem encountered in patients undergoing maintenance hemodialysis (MHD). This issue could be attributed to mechanical stimulation/damage to vessel walls from central vein catheterization and catheter indwelling, as well as the high-flow status of a functional fistula [1,2]. Although it is usually asymptomatic, symptomatic CVOD manifests as swelling and pain in the ipsilateral limb, neck, face or head and undermines the integrity of the hemodialysis circuit, thereby compromising adequate dialysis [2,3]. Percutaneous transluminal balloon angioplasty (PTA) with selective stenting (PTS) has recently become the primary approach for treating symptomatic CVOD. Although PTA alone for treating symptomatic CVOD has a technical success rate of 70–100%, patients resistant to PTA continue to suffer from refractory CVOD; for these refractory cases, stent deployment is often the next step [3–6]. However, the efficacy of stenting in treating CVOD remains controversial [5–8], and outcomes such as vascular access (VA) longevity and survival rates in this population are scarce. This leads to difficulties in clinical decision-making, e.g., whether to recanalize the occlusive hemodialysis circuit with a stenting strategy or to ligate the circuit to relieve swelling symptoms while depleting new blood vessels to establish a new VA [4,9,10]. Therefore, this study was designed to determine long-term outcomes in MHD patients with refractory CVOD receiving PTS treatment.

## Patients and methods

### Study population

MHD patients who received their first successful PTS treatment due to symptomatic CVOD between September 2016 and September 2019 at the Department of Nephrology in a major tertiary hospital (Chengdu, China) were consecutively included in this retrospective study. MHD-related CVOD was confirmed by angiography, which revealed >50% stenosis or complete occlusion of the internal jugular vein, subclavian vein, brachiocephalic vein, or superior vena cava (SVC) [2,6]. Following the European Society for Vascular Surgery guidelines [11], PTS was performed for patients with refractory CVOD presenting immediate elastic retraction or residual stenosis ≥30% or >10% in the left brachiocephalic vein near the SVC region after balloon angioplasty or recurrent cases within 3–6 months after angioplasty. The exclusion criteria for the patients were as follows: (1) had preexisting stents in central veins for any reason and (2) refused to consent to the study.

The study was performed in accordance with the principles of the Declaration of Helsinki and was approved by the Ethics Committee of West China Hospital, Sichuan University (No. KS2019093). Written or verbal informed consent was obtained from all participants.

### Clinical data and interventional procedure

Demographic data and clinical characteristics with respect to coexisting conditions, laboratory examinations, imaging data and operation records were acquired through the electronic medical records system. Central vein stenosis (CVS) was scored *via* a semiquantitative method based on radiological imaging as previously reported [6], and each vein was rated on a scale of 0–3, with a total possible maximum score of 12. The vein with the severest lesions (with the highest CVS score) was considered the main vein affected by CVOD.

Blunt and/or sharp recanalization techniques were utilized to recanalize the occlusion lesions. The blunt recanalization technique refers to the use of the soft end of the guide wire supported by the catheter or vessel sheath to pass through the occlusion segment. The sharp recanalization technique, which uses interventional instruments with superior flexibility and support force [e.g., the guidewire hard head, RUPS-100 puncture kit (Cook, Bloomington, USA), and atrial septal puncture kit (St. Jude Medical, St. Paul, USA)] to traverse occlusions, is applied when the blunt recanalization technique fails. The percutaneous SVC puncture technique, with years of experience in the clinic, was employed specifically for complex lesions involving long-segment SVC occlusion. The cutaneous puncture site is 0.5–1.0 cm lateral–inferior to the clavicular head of the sternocleidomastoid muscle [8]. A successful SVC puncture facilitates the division of long-segment SVC occlusions into upper and lower relatively short sections, thereby reducing the degree of difficulty with recanalization [6].

Following successful recanalization, sequential balloon (Cordis, CA, USA) dilation was performed with inflation pressures of 4–10 atm. Shape Memory Alloy Recoverable Technology (SMART) bare metal stents (Cordis, CA, USA), Fluency covered metal stents (Bard, NJ, USA), Viabahn covered metal stents (Gore, Newark, USA), or Z-bare metal stents (Antai, Beijing, China) were implanted by two operators based on the diameter, length, angulation of lesions, inventory of stent size, and patient preferences. The protocol for selecting the stent size is as follows: the diameter of the stent should be more than 10–20% of the diameter of the adjacent vessel, and the length of the stent should be sufficient to extend its two ends into the normal vessel adjacent to the lesion [6].

### Follow-up and outcome definitions

Patients were followed up in the outpatient department, hemodialysis centers, through phone calls, or electronic medical records until death or were censored for receiving kidney transplants or switching to peritoneal dialysis. The study period ranged from the first successful PTS treatment to the last follow-up or the date of death. The primary endpoints were recurrence of symptomatic CVOD (rCVOD), VA abandonment, cardiovascular death, and all-cause death. Typically, rCVOD can develop in stented vessels or new blood vessels in the central vein system.

Consistent with published definitions [12,13], the primary patency rate (PPR) in this study was defined as the proportion of patients without any reintervention for maintaining the patency of central veins during the period from the first successful PTS. The assisted patency rate (APR) of the central vein/VA was the proportion of patients who experienced central vein/VA patency, with or without reintervention manipulations during the study period. VA abandonment was defined as a fistula, graft, or catheter that could not provide adequate flow for hemodialysis and/or was deemed unsafe for the patient, and the associated problem could not be rectified by any intervention. Specifically, planned removal of a functional catheter that was used as a transitioning VA before the fistula matured was not considered VA abandonment.

### Statistical analysis

Continuous variables are presented as the means ± standard deviations (SDs) for normally distributed variables or medians with interquartile ranges (IQRs) for nonnormally distributed variables. Categorical variables are presented as numbers and corresponding percentages. Comparisons between the rCVOD and non-rCVOD groups were conducted *via* two-sample *t* tests or Wilcoxon rank sum tests for quantitative variables and chi-square tests for categorical variables. The life-table method was used to calculate the patency rates, and survival curves were generated *via* the Kaplan–Meier method. Univariate and multivariate logistic regression analyses were conducted to explore risk factors for VA abandonment or mortality. Variables with *p* < 0.1 in the univariate analysis and those considered clinically relevant to the dependent variable or that were aimed to clarify in this study were entered into the multivariate regression models, with odds ratios (ORs) and 95% confidence intervals (CIs) reported. The Hosmer–Lemeshow test and classification table were employed to explore the goodness-of-fit and reliability of the relatively optimal models, with *p* > 0.05 regarded as an acceptable model. The level of significance was set to a two-sided *p* value of <0.05. The data were analyzed *via* SPSS version 23.0 (IBM, NY, USA).

## Results

**Figure 1.**
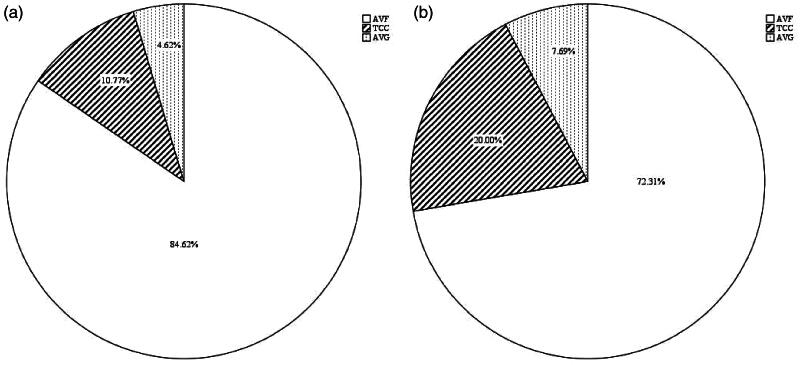
Types of permanent vascular access at enrollment (A) and at the end of the study (B). AVF, arteriovenous fistula; TCC, tunneled cuffed catheter; AVG, arteriovenous graft.

Seventy-seven MHD patients who received their first PTS treatment due to symptomatic CVOD were reviewed. Five patients were excluded for failed PTS treatments: three experienced sharp recanalization failure, and two experienced severe complications during recanalization, including one cardiac tamponade and one acute hemothorax. The case of cardiac tamponade was rescued by emergency perforation repair and thrombus removal of the right atrium, and the acute hemothorax case was rescued by inserting a spring coil to embolize the puncture path and stop bleeding [6]. There were no procedure-related deaths. Among the 72 patients with successful PTS treatment (anatomical and clinical success), 7 dropped out because of missing information regarding treatments for rCVOD at 13 (8.0–17.5) months. Finally, a total of 65 patients (60.9% male) aged 61.5 ± 13.5 years were included, with one patient censored for successful kidney transplantation at 61 months. The mean dialysis vintage was 54.7 ± 39.1 months. In terms of permanent VA history, 45 (69.2%), 12 (18.5%), 5 (7.7%), 2 (3.1%), and 1 (1.5%) patients used arteriovenous fistula (AVF), AVF + tunneled cuffed catheter (TCC), TCC, arteriovenous graft (AVG)+ TCC, and AVG, respectively. The main VA used for dialysis before PTS treatment was AVF (84.6%) (Figure 1A). The main veins affected by CVOD were the subclavian vein, brachiocephalic vein, and SVC in 16 (24.6%), 40 (61.5%), and 9 (13.8%) patients, respectively, and the median CVS was 8.0 (6.0–9.0) points. The stents implanted were Fluency covered stents (37 patients, 56.9%), SMART bare stents (29 patients, 44.6%), Viabahn covered stents (5 patients, 7.7%) and Z-bare metal stents (2 patients, 3.1%) (Table 1).

**Table 1.** Characteristics and outcomes of 65 patients on maintenance hemodialysis according to recurrence of Central venous occlusive disease.

Variable	Total	rCVOD	Non-rCVOD	*p*
No. of patients	65	32	33	
Male no. (%)	34 (52.3)	20 (62.5)	14 (42.4)	0.105
Age (year)	61.5 ± 13.5	62.1 ± 11.0	60.9 ± 15.6	0.718
Diabetes Mellitus no. (%)	18 (27.7)	8 (25.0)	10 (30.3)	0.633
Coronary heart disease no. (%)	22 (33.8)	11 (34.4)	11 (33.3)	0.929
Dialysis vintage (month)	54.7 ± 39.1	63.1 ± 40.9	46.7 ± 35.9	0.091
Hemoglobin (g/L)	108.6 ± 17.4	111.6 ± 19.8	105.6 ± 14.5	0.166
Albumin (g/L)	42.8 (40.4–44.9)	43.3 (42.2–45.0)	41.4 (39.1–44.5)	0.015
Main vein of CVOD				0.162
subclavian vein no. (%)	16 (24.6)	11 (34.4)	5 (15.2)	–
brachiocephalic vein no. (%)	40 (61.5)	18 (56.3)	22 (66.7)	–
superior vena cava no. (%)	9 (13.8)	3 (9.4)	6 (18.2)	–
Stent type no. (%)				
SMART bare metal stent	29 (44.6)	15 (46.9)	14 (42.4)	0.718
Fluency covered metal stent	37 (56.9)	18 (56.3)	19 (57.6)	0.914
CVS score	8.0 (6.0–9.0)	7.0 (5.5–9.0)	8.0 (6.0–10.0)	0.548
VA abandonment no. (%)	10 (15.4)	6 (18.8)	4 (12.1)	0.692
Cardiovascular death no. (%)	6 (9.2)	2 (6.3)	4 (12.1)	0.697
All-cause death no. (%)	17 (26.2)	6 (18.8)	11 (33.3)	0.181
Follow-up time (month)	40.0 (20.0–54.0)	48.0 (30.5–60.5)	25.0 (15.0–51.0)	0.013

Abbreviations: rCVOD: recurrence of central venous occlusive disease; CVOD, central venous occlusive disease; CVS, central vein stenosis; VA, vascular access; PPR, primary patency rate.

**Figure 2.**
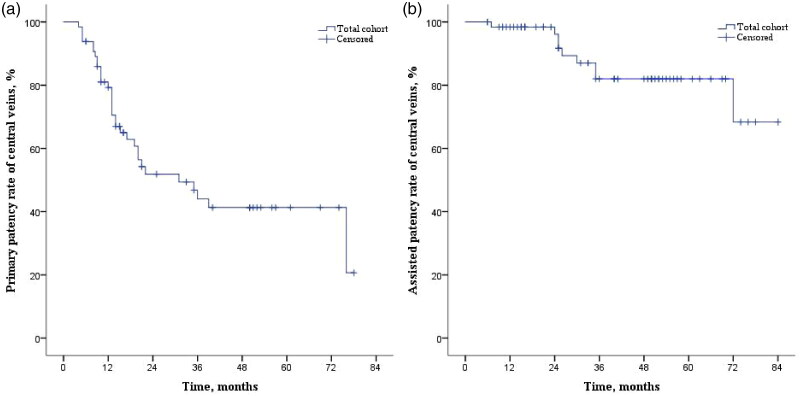
Curves illustrating the primary (A) and assisted (B) patency rates of Central veins among study patients after the first successful stenting. rCVOD, recurrent symptomatic CVOD. Footnote to Figure 2A

**Figure 3.**
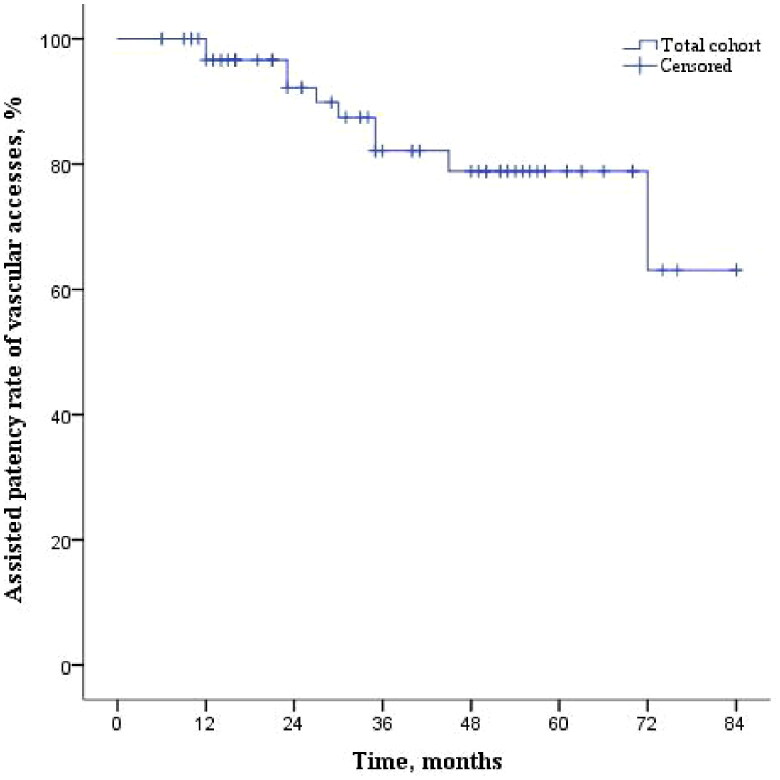
Curves illustrating the assisted patency rate of vascular accesses among study patients after the first successful stenting. Footnote to Figure 3

During 40.0 (20.0–54.0) months of follow-up, symptomatic CVOD recurred in 32 (49.2%) patients, with 30 recurrent cases resulting from in-stent restenosis (lumen stenosis >50%). The PPR and APR of the central veins at 12, 24, 36, 48, and 60 months were 81%, 52%, 47%, 41%, and 41% (Figure 2A) and 98%, 98%, 82%, 82%, and 82% (Figure 2B), respectively. For reintervention, 51 secondary PTA treatments with 34 stenting procedures were performed for patients with rCVOD. VA abandonment was noted in 10 patients (15.4%), and the VAs used at the end of the study are shown in Figure 1B. The APRs of VAs at 12, 24, 36, 48, and 60 months after stenting were 100%, 92%, 82%, 79%, and 79%, respectively (Figure 3). There was no significant difference in the patencies of central veins between patients with covered stents and those with non-covered (bare metal) stents (Supplementary Figure 1). All-cause mortality was 26.2% (17 patients), among whom 6 patients (9.2%) died from cardiovascular causes, including 4 deaths due to congestive heart failure, one death due to stroke and one death due to severe dialysis-related hypotension.

**Table:** 

Months	0-12	12-24	24-36	36-48	48-60	60-72	72-84
No. of cases entering the time interval	65	47	22	17	14	5	3
No. of events at the interval	12	15	2	2	0	0	1 Footnote to Figure 2B

**Table:** 

Months	0-12	12-24	24-36	36-48	48-60	60-72	72-84
No. of cases entering the time interval	65	58	44	32	28	12	6
No. of events at the interval	1	0	7	0	0	0	1

**Table:** 

Months	0-12	12-24	24-36	36-48	48-60	60-72	72-84
No. of cases entering the time interval	65	59	41	29	24	10	5
No. of access abandonment at the interval	0	4	4	1	0	0	1

As shown in Table 2, the number of secondary PTAs (OR = 8.253, 95% CI 1.405–48.468, *p* = 0.019) was an independent risk factor for VA abandonment, whereas coexisting coronary heart disease (OR = 0.049, 95% CI 0.002–0.956, *p* = 0.047) and secondary PTS treatment (OR = 0.089, 95% CI 0.008–0.992, *p* = 0.049) were found to be independent protective factors. Moreover, subsequent PTSs (OR = 0.104, 95% CI 0.011–0.947, *p* = 0.045) and elevated serum levels of albumin (OR = 0.801, 95% CI 0.665–0.965, *p* = 0.019) were significantly associated with decreased all-cause mortality (Table 3).

**Table 2.** Risk factors for the vascular access abandonment in logistic regression analysis.

Variable	OR (univariate)	95%CI (univariate)	*p* (univariate)	OR (multivariate)	95%CI (multivariate)	*p* (multivariate)
Age (/1 year)	0.981	0.933–1.030	0.436	1.051	0.971–1.139	0.219
Diabetes Mellitus (yes = 1)	0.248	0.029–2.119	0.203	0.111	0.006–1.907	0.130
Coronary heart disease (yes = 1)	0.180	0.021–1.524	0.116	0.049	0.002–0.956	0.047
Hemoglobin (/1 g/L)	0.976	0.939–1.015	0.227	0.951	0.897–1.008	0.093
No. of PTA (/1)	1.354	0.760–2.414	0.304	8.253	1.405–48.468	0.019
No. of PTS (/1)	0.954	0.390–2.335	0.918	0.089	0.008–0.992	0.049
Follow-up time to access abandonment (/1 month)	0.985	0.952–1.019	0.388	0.957	0.904–1.014	0.134
Constant				12.050	–	0.428

Abbreviations: OR: odds ratio; CI: confidence interval; PTA: percutaneous transluminal angioplasty; PTS, percutaneous transluminal angioplasty with stenting.

Hosmer‒Lemeshow *χ*2 = 3.794, *p =* 0.803, percentage correct 86.2%.

**Table 3.** Risk factors for all-cause death in logistic regression analysis.

Variable	OR (univariate)	95%CI (univariate)	*p* (univariate)	OR (multivariate)	95%CI (multivariate)	*p* (multivariate)
Age (/1 year)	1.055	1.004–1.109	0.034	1.056	0.979–1.139	0.159
Diabetes Mellitus (yes = 1)	4.875	1.473–16.134	0.009	1.496	0.281–7.967	0.637
Coronary heart disease (yes = 1)	4.286	1.335–13.755	0.014	2.591	0.525–12.775	0.242
Albumin (/1 g/L)	0.853	0.742–0.980	0.025	0.801	0.665–0.965	0.019
CVS score (/1 point)	0.967	0.779–1.201	0.762	0.905	0.686–1.194	0.481
No. of PTA (/1)	0.674	0.347–1.308	0.244	3.343	0.838–13.346	0.087
No. of PTS (/1)	0.288	0.086–0.969	0.044	0.104	0.011–0.947	0.045
Follow-up time to all-cause death (/1 month)	0.973	0.945–1.001	0.055	0.983	0.950–1.018	0.343
Constant				254.668	–	0.117

Abbreviations: OR: odds ratio; CI: confidence interval; CVS, central vein stenosis; PTA, percutaneous transluminal angioplasty; PTS, percutaneous transluminal angioplasty with stenting.

Hosmer‒Lemeshow *χ*2 = 7.170, *p* = 0.411, percentage correct 80.0%.

## Discussion

The present study supported the use of a stenting strategy in treating CVOD based on the potencies of the treated lesions. A recent meta-analysis of eight studies (six retrospective studies) encompassing 473 patients with symptomatic CVOD indicated that PTS did not result in a superior PPR or APR to PTA alone during a 24-month follow-up [7]. However, the methodology in the meta-analysis was biased because most patients were given PTS treatment because PTA was not effective; thus, the PTS group might have suffered more severe lesions than the PTA group at baseline. In the case of refractory CVOD that is resistant to PTA, the choice faced by patients is not to choose between PTA and PTS but rather to choose whether to deploy a stent or terminate endovascular therapy and resort to surgical treatments. Traditional surgical reconstructions [14,15], such as axillary–axillary vein crossover and jugular vein bypass, although having an acceptable patency rate of 80% at 12 months, are frequently impossible because of the lack of suitable anatomy in the central veins. Hemodialysis Reliable Outflow (HeRO) grafting is a relatively new surgical strategy that provides internal bypass through stenotic lesions. However, placing HeRO grafts for treating recurrent CVOD has shown similar reintervention and patency rates to those of stenting, as observed in a recent comparative study [16].

In terms of the device used for stenting, dedicated venous stents were not studied here because such stents had not yet been introduced into the hospital at enrollment. Typically, stenting in the venous system has evolved from experiences in arterial settings. Given differences in the pathophysiology of arterial stenosis and venous obstruction, an ideal venous stent has specialized requirements with respect to radial force, flexibility, size, durability, etc., differing from those needed in the arterial system [17]. In this context, multiple dedicated stents for the venous system have been developed and have exhibited comparable patency (PPR 95% and APR 100% at 1 year) and clinical outcomes to non-dedicated stents in treating iliofemoral obstruction [18]. For hemodialysis patients with upper thoracic CVOD, three studies used dedicated venous stents, including sinus/sinus-XL stents (*n* = 34), Abre stents (*n* = 15), and Vici stents (*n* = 18), which presented varied PPRs (61.8–94.4%) at 12 months [19–21]. Although the comparative study revealed a significantly greater 12-month PPR in the sinus venous stent group than in the conventional bare stent (Epic, SMART, or Protege GPS stents) group (61.8% *vs.* 32.6%; *p* = 0.008) [19], the PPRs were not superior to those in the current study, which used 4 types of non-dedicated venous stents, or several previous studies utilizing covered stents such as the Viabahn and Excluder stents [22]. The difference in the efficacy of current venous stents may result from individual conditions, lesion characteristics, the mechanical configuration and material composition of the stents, and post-procedure management. Thus, the application of dedicated venous stents in treating hemodialysis-related CVOD remains challenging, and the existing limited evidence necessitates further large-scale, high-quality investigations.

Furthermore, this study revealed superior VA longevity compared with that of a previous study, which included 73 patients with symptomatic CVOD treated by PTA or PTS and reported a cumulative VA survival of 32–38% at 24 months [9]. The multivariate analysis revealed a significant inverse correlation between subsequent PTAs with VA longevity and a significant positive correlation between subsequent PTSs and VA longevity, although statistical significance was not detected in the univariate analysis, possibly due to the small sample size. This inverse correlation might be because the increased number of subsequent PTAs indicated frequent rCVOD, which was directly associated with VA dysfunction and VA abandonment, and this positive correlation might suggest that PTS treatment was more efficient in maintaining the patency of the hemodialysis circuit than was PTA alone. Therefore, the better VA survival in this study than in the previous study [9] might be partly explained by the 100% PTS application rate.

In addition, the present refractory CVOD cohort did not have significantly greater mortality than the general hemodialysis population did [23,24], and rCVOD did not predict a poor survival rate. Additionally, subsequent PTSs were shown to be an independent protective factor for patient survival. This could be attributed to the above benefits of PTS treatment in maintaining the hemodialysis circuit, thereby contributing to adequate dialysis and improved outcomes [25,26], and attributed to the few adverse events related to the procedure. These positive survival data highlighted a stenting strategy for recanalizing dysfunctional hemodialysis circuits for long-term dialysis in MHD patients.

The present study has the strengths of a long follow-up period and the use of outcome indicators, such as VA longevity and patient survival, to clarify the significance of stenting therapy in refractory CVOD. Nevertheless, several limitations in the study cannot be ignored. First, although the sample size was larger than those in most similar studies on the rare population with refractory CVOD [5,8,27], the population cohort was small. Second, the variety of stents used in this study could introduce heterogeneity in the results. Third, the retrospective design effected inherent bias, as precise information on relevant events, such as anticoagulant medication, dialysis prescriptions, and reinterventions administered at other clinics during follow-up, might not be obtained; this deviation might have caused certain differences in the APRs.

## Conclusions

The present study suggested that PTS treatment was a viable and effective strategy for MHD patients suffering refractory CVOD, based on the prominent patency rates of treated veins and the significant correlations of subsequent PTSs with improved VA longevity and patient survival. Additional studies are needed to address this issue and establish an optimized PTS protocol.

## Supplementary Material

Figure S1 A.jpg

Figure S1 B.jpg

## Data Availability

All the data generated or analyzed during this study are included in this article. Further inquiries can be directed to the corresponding author.

## References

[1] Kundu S. Review of central venous disease in hemodialysis patients. J Vasc Interv Radiol. 2010;21(7):963–968. doi: 10.1016/j.jvir.2010.01.044. 20418112

[2] Krishna VN, Eason JB, Allon M. Central venous occlusion in the hemodialysis patient. Am J Kidney Dis. 2016;68(5):803–807. doi: 10.1053/j.ajkd.2016.05.017. 27492146

[3] Echefu G, Stowe I, Lukan A, et al. Central vein stenosis in hemodialysis vascular access: clinical manifestations and contemporary management strategies. Front Nephrol. 2023;3:1280666. doi: 10.3389/fneph.2023.1280666. 38022724 PMC10664753

[4] Saleh M, Ali H, Elbadawy A, et al. Balloon angioplasty with selective stenting strategy in treatment of hemodialysis related central vein occlusive lesions. Int Angiol. 2017;36(5):462–466. doi: 10.23736/S0392-9590.17.03817-2. 28541018

[5] Maya ID, Saddekni S, Allon M. Treatment of refractory central vein stenosis in hemodialysis patients with stents. Semin Dial. 2007;20(1):78–82. doi: 10.1111/j.1525-139X.2007.00246.x. 17244127

[6] Yu Y, Xiong Y, Li T, et al. Risk factors for in-stent restenosis in maintenance hemodialysis patients with central venous occlusive disease and biomechanical assessment of stents. J Vasc Access. 2024;25(3):943–952. doi: 10.1177/11297298221139640. 36540050

[7] Wu TY, Wu CK, Chen YY, et al. Comparison of percutaneous transluminal angioplasty with stenting for treatment of central venous stenosis or occlusion in hemodialysis patients: a systematic review and meta-analysis. Cardiovasc Intervent Radiol. 2020;43(4):525–540. doi: 10.1007/s00270-019-02383-7. 31900506

[8] Xiong Y, Yu Y, Cui T. Angled, long-segment central venous occlusion in a hemodialysis patient recanalized by a novel “two-step” strategy based on percutaneous superior vena cava puncture. Ther Apher Dial. 2021;25(5):712–713. doi: 10.1111/1744-9987.13591. 33006430

[9] Bakken AM, Protack CD, Saad WE, et al. Long-term outcomes of primary angioplasty and primary stenting of central venous stenosis in hemodialysis patients. J Vasc Surg. 2007;45(4):776–783. doi: 10.1016/j.jvs.2006.12.046. 17398386

[10] Quaretti P, Galli F, Moramarco LP, et al. Stent grafts provided superior primary patency for central venous stenosis treatment in comparison with angioplasty and bare metal stent: a retrospective single center study on 70 hemodialysis patients. Vasc Endovascular Surg. 2016;50(4):221–230. doi: 10.1177/1538574416639149. 27097842

[11] Schmidli, Jürg, Widmer, Matthias K, Basile, Carlo, et al. Editor’s choice- vascular access: 2018 clinical practice guidelines of the European Society for Vascular Surgery (ESVS). Eur J Vasc Endovasc Surg 2018; 55(6): 757–818. doi: 10.1016/j.ejvs.2018.02.001. 29730128

[12] Lee T, Mokrzycki M, Moist L, North American Vascular Access Consortium., et al. Standardized definitions for hemodialysis vascular access. Semin Dial. 2011;24(5):515–524. doi: 10.1111/j.1525-139X.2011.00969.x. 21906166 PMC3999346

[13] Shenoy S, Allon M, Beathard G, et al. Clinical trial end points for hemodialysis vascular access: background, rationale, and definitions. Clin J Am Soc Nephrol. 2018;13(3):490–494. doi: 10.2215/CJN.13321216. 29487092 PMC5967685

[14] Dammers R, de Haan MW, Planken NR, et al. Central vein obstruction in hemodialysis patients: results of radiological and surgical intervention. Eur J Vasc Endovasc Surg. 2003;26(3):317–321. doi: 10.1053/ejvs.2002.1943. 14509897

[15] Bhatia DS, Money SR, Ochsner JL, et al. Comparison of surgical bypass and percutaneous balloon dilatation with primary stent placement in the treatment of central venous obstruction in the dialysis patient: one-year follow-up. Ann Vasc Surg. 1996;10(5):452–455. doi: 10.1007/BF02000591. 8905064

[16] Proksch DM, Rodriguez LE, Rathore A, et al. A comparison of stenting versus hemodialysis reliable outflow graft for hemodialysis patients with recurrent central venous obstructions. J Vasc Surg Venous Lymphat Disord. 2021;9(5):1136–1144. doi: 10.1016/j.jvsv.2021.01.001. 33453441

[17] Shamimi-Noori SM, Clark TWI. Venous stents: current status and future directions. Tech Vasc Interv Radiol. 2018;21(2):113–116. doi: 10.1053/j.tvir.2018.03.007. 29784119

[18] Majeed GM, Lodhia K, Carter J, et al. A systematic review and meta-analysis of 12-month patency after intervention for iliofemoral obstruction using dedicated or non-dedicated venous stents. J Endovasc Ther. 2022;29(3):478–492. doi: 10.1177/15266028211057085. 34758673 PMC9096580

[19] Akkakrisee S, Hongsakul K. Venous stent versus conventional stent for the treatment of central vein obstruction in hemodialysis patients: a retrospective study. Acta Radiol. 2022;63(1):59–66. doi: 10.1177/02841851211005163. 33779305

[20] Aronhime S, Balan S, Timokhin A, et al. Early experience with the Abre venous stent for central venous stenoses and occlusions in hemodialysis patients. J Vasc Access. 2024;25(6):1961–1966. doi: 10.1177/11297298231193893. 37622463

[21] Tan GM, Chi KWK, Yan BPY. Mid-term results of a novel dedicated venous stent for the treatment of chronic thoracic central vein obstruction of benign aetiology. Eur J Vasc Endovasc Surg. 2019;57(3):417–423. doi: 10.1016/j.ejvs.2018.10.009. 30404722

[22] Kitrou P, Katsanos K, Karnabatidis D. Management of central venous stenoses and occlusions. Cardiovasc Intervent Radiol. 2023;46(9):1182–1191. doi: 10.1007/s00270-023-03461-7. 37460644 PMC10471665

[23] Nishi H, Wang J, Onishi Y, et al. Infectious risk and variability of hemoglobin level in patients undergoing hemodialysis. Kidney Int Rep. 2023;8(9):1752–1760. doi: 10.1016/j.ekir.2023.06.004. 37705913 PMC10496019

[24] Karaboyas A, Xu H, Morgenstern H, et al. DOPPS data suggest a possible survival benefit of renin angiotensin-aldosterone system inhibitors and other antihypertensive medications for hemodialysis patients. Kidney Int. 2018;94(3):589–598. doi: 10.1016/j.kint.2018.03.013. 29908836

[25] Hong WP, Lee YJ. The association of dialysis adequacy, body mass index, and mortality among hemodialysis patients. BMC Nephrol. 2019;20(1):382. doi: 10.1186/s12882-019-1570-0. 31640580 PMC6805311

[26] Aghsaeifard Z, Zendehdel A, Alizadeh R, et al. Chronic hemodialysis: evaluation of dialysis adequacy and mortality. Ann Med Surg (Lond). 2022;76:103541. doi: 10.1016/j.amsu.2022.103541. 35495410 PMC9052277

[27] Keller EJ, Gupta SA, Bondarev S, et al. Single-center retrospective review of radiofrequency wire recanalization of refractory central venous occlusions. J Vasc Interv Radiol. 2018;29(11):1571–1577. doi: 10.1016/j.jvir.2018.06.017. 30293732

