# Supplementary figures and images for: Long-term outcomes of refractory central venous occlusive disease treated by stent deployment in patients undergoing maintenance hemodialysis
Source: Ren Fail. 2025 Feb 16;47(1):2463579. doi: 10.1080/0886022X.2025.2463579 (PMC11834813; doi:10.1080/0886022X.2025.2463579)

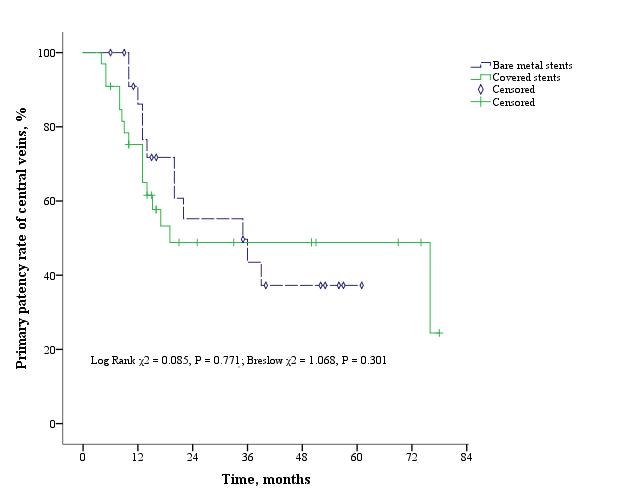

Supplement: Figure S1 A.jpg [file IRNF_A_2463579_SM2263.jpg]

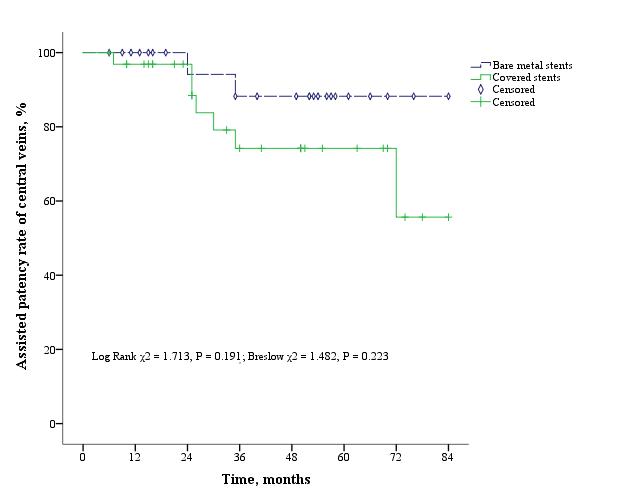

Supplement: Figure S1 B.jpg [file IRNF_A_2463579_SM2262.jpg]
